# Laser-Engineered Interfacial Dielectrophoresis-Aligned Nanowire Networks for Transparent Electromagnetic Interference-Shielding Films

**DOI:** 10.1021/acsnano.5c13772

**Published:** 2025-12-16

**Authors:** Jungang Zhang, Venkatarao Selamneni, Bhavani Prasad Yalagala, Benjamin King, Jiaoran Wang, Luvsanbat Khurelbaatar, Carlos García Núñez, Mahmoud Wagih, Morteza Amjadi, Hadi Heidari

**Affiliations:** † Microelectronics Laboratory (meLAB), James Watt School of Engineering, 150841 University of Glasgow, Glasgow G12 8QQ, U.K.; ‡ Biomedical Soft Robotics Group, James Watt School of Engineering, 3526 University of Glasgow, Glasgow G12 8QQ, U.K.; § Green RF-Enabled Electronics Laboratory, James Watt School of Engineering, 3526 University of Glasgow, Glasgow G12 8QQ, U.K.

**Keywords:** nanowires, interfacial-dielectrophoresis, noncontact laser post-treatment, electromagnetic interference shielding, flexible and transparent electronic devices

## Abstract

Nanowires (NW) hold substantial promise for high-performance electronics; however, the lack of programmable, deterministic control and alignment strategies limits their seamless integration onto flexible target substrates, posing challenges to manufacturing reliability, efficiency, and scalability. Herein, we propose a scalable and adaptable interfacial dielectrophoresis (i-DEP) method for precise translational and rotational (0–150°) manipulation of NWs on thin polymer films. A subsequent noncontact picosecond laser post-treatment is introduced to effectively enhance electrical and optical properties by welding NW junctions through controlled thermal diffusion and localized-field confinement. The laser-welded silver nanowires (AgNW) network has attained ∼46× reduction in sheet resistance and a 10% enhancement in transmittance. The i-DEP aligned, laser-treated ultrathin AgNW/polyimide film achieves 35 dB electromagnetic interference shielding effectiveness, demonstrating over 1000× improvement compared to randomly oriented drop-cast AgNW networks. The additional shielding contribution arises from the capacitively coupled interwire network in the predefined aligned-NWs structure, realizing performance beyond that attainable by conductivity alone. This work presents a laser-engineered, i-DEP-aligned NW network that reinforces interfacial NW network quality and provides a systematic fabrication and optimization strategy for advancing wireless, flexible, and high-performance transparent electronic devices.

## Graphical abstract

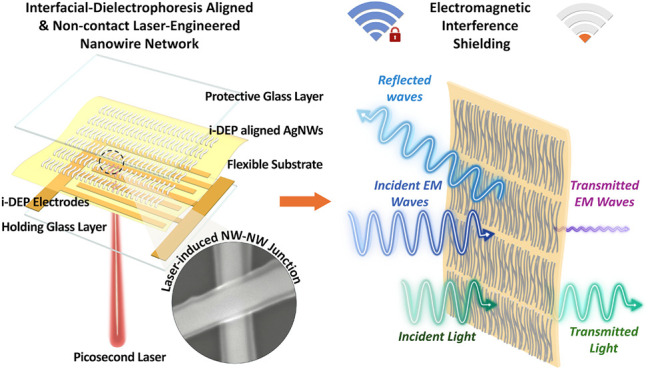

## Introduction

Flexible and transparent conductive films/electrodes, which use metallic NWs such as copper (Cu), silver (Ag), and gold (Au),^1–4^ are among the building blocks in electromagnetic interference (EMI) shielding materials for upcoming technologies, including 6G wireless communication networks, Wi-Fi systems, radar, and biotelemetry.^5^ Among these materials, AgNWs have been investigated for diverse applications owing to their excellent electrical conductivity, high optical transparency, and strong mechanical compliance.^6,7^ Numerous synthesis methods have been developed for NW-based composite films, including physical and chemical routes such as chemical vapor transport,^8^ solvothermal, polyol, and hydrothermal techniques, facilitating the growth of high-quality, high-yield NWs.^9,10^ Nevertheless, precise postgrowth positional alignment and control of the NWs, compatible with both single and multilayered configurations over large-area thin films, persist as a critical barrier for the fabrication and integration of high-performance NW-based devices. Although various techniques, including Langmuir–Blodgett deposition, photolithography, contact printing, and microfluidic flow, have been explored for NW manipulation, they remain limited in achieving high-precision and simultaneous control over NW position and orientation.^11^ Additionally, state-of-the-art methods are often time-consuming and costly, restricting their adaptation to a wide range of high-performance NW-based electronics.^12^

An alternative solution is dielectrophoresis (DEP), which utilizes a nonuniform electric field to induce polarization in nanostructures, including NWs, nanoparticles, and nanotubes.^13,14^ The polarized nanostructures interact with the electric field gradient in fluid, enabling precise control over their assembly, positioning, and orientation at specific patterns, assisted by predefined electrodes.^15^ Numerous efforts have advanced the DEP technique toward highly selective manipulation through the optimization of frequency, voltage, electrode geometry, fluid type, dielectric properties, nanostructure concentration, and dimension.^16,17^ However, several key hurdles require immediate attention to enable widespread adoption of DEP in device manufacturing, including: (i) selective alignment of different types of NWs on emerging flexible substrates to facilitate seamless integration onto thin films, (ii) precise control of NW alignment with full degrees of freedom in both translational and rotational orientations, (iii) a simple, transfer-free process for NW assembly on independent interfaces with isolated, sustainable, and reusable electrodes, and (iv) rapid, scalable, and cleanroom-free fabrication of DEP electrodes.

Herein, we report a scalable and efficient, laser-engineered i-DEP method for precise alignment and assembly of NWs with tailored optical and electrical properties onto ultrathin, conformable polymeric interfaces (Figure 1). This method facilitates spatially reconfigurable NW assembly beyond wafer-scale limitations and allows for the isolation of both electrodes and aligned NW films without cross-contamination. Notably, i-DEP electrodes were fabricated via direct patterning with an ultrashort-pulsed laser (Figure 1a), eliminating reliance on conventional photolithography processes and enabling low-cost electrode fabrication with high throughput, material versatility, and design flexibility. Building on the i-DEP strategy, highly controllable and precise assembly of AgNWs was achieved on flexible and semitransparent polyimide (PI) films across predefined electrodes, demonstrating discrete angular manipulation (0–150°) and combined rotational-translational positioning. Additionally, large-area (40 cm^2^ and 80 cm^2^) AgNW/PI films were fabricated for EMI shielding applications. However, a fundamental trade-off persists between optical transmittance and sheet resistance (Rs) in the NW conductive film. Specifically, increasing the NW density or layers enhances electrical conductivity but degrades transparency. To address this unmet challenge, an optimized noncontact laser post-treatment (Figure 1b) was applied to a single-layer i-DEP aligned AgNW network (submicron thickness), resulting in ∼46× reduction in Rs (from 1682 Ω/sq to 37 Ω/sq) while maintaining high transmittance with a slight improvement (∼10%). The i-DEP fabricated, laser-welded AgNW/PI films exhibit exceptional flexibility and optical transparency (83.1%) while delivering robust EMI shielding performance with a shielding effectiveness (SE) exceeding 35 dB across the 2.2–6 GHz midband spectrum. This demonstrates that the proposed laser-sintered i-DEP-aligned NWs achieve up to a 1000× enhancement in broadband radio frequency (RF) response compared to random AgNW networks, while offering scalability and mechanical flexibility (Figure 1c). Particularly, the i-DEP aligned NW network, featuring structural nanoscale gaps that form a capacitively coupled interwire network, facilitates local electric-field coupling and displacement-current flow, thereby significantly enhancing EMI shielding performance beyond the contribution of conductivity alone.^18–20^ The resulting ultrathin, flexible, and transparent EMI shielding film operates across the midband frequencies typical of most communication systems,^18,21^ supporting secure wireless biotelemetry and mitigating risks associated with long-term EM exposure. In addition, real-time EMI shielding performance was validated by transmitting a 5 GHz Wi-Fi signal in an anechoic chamber, showing an approximate 15 dB reduction in the receiver signal strength indicator (RSSI). These results highlight the substantial potential of laser-treated, i-DEP aligned AgNW films for EMI protection in conformal and optically transparent, wireless wearable and/or implantable electronic devices.

**Figure 1:**
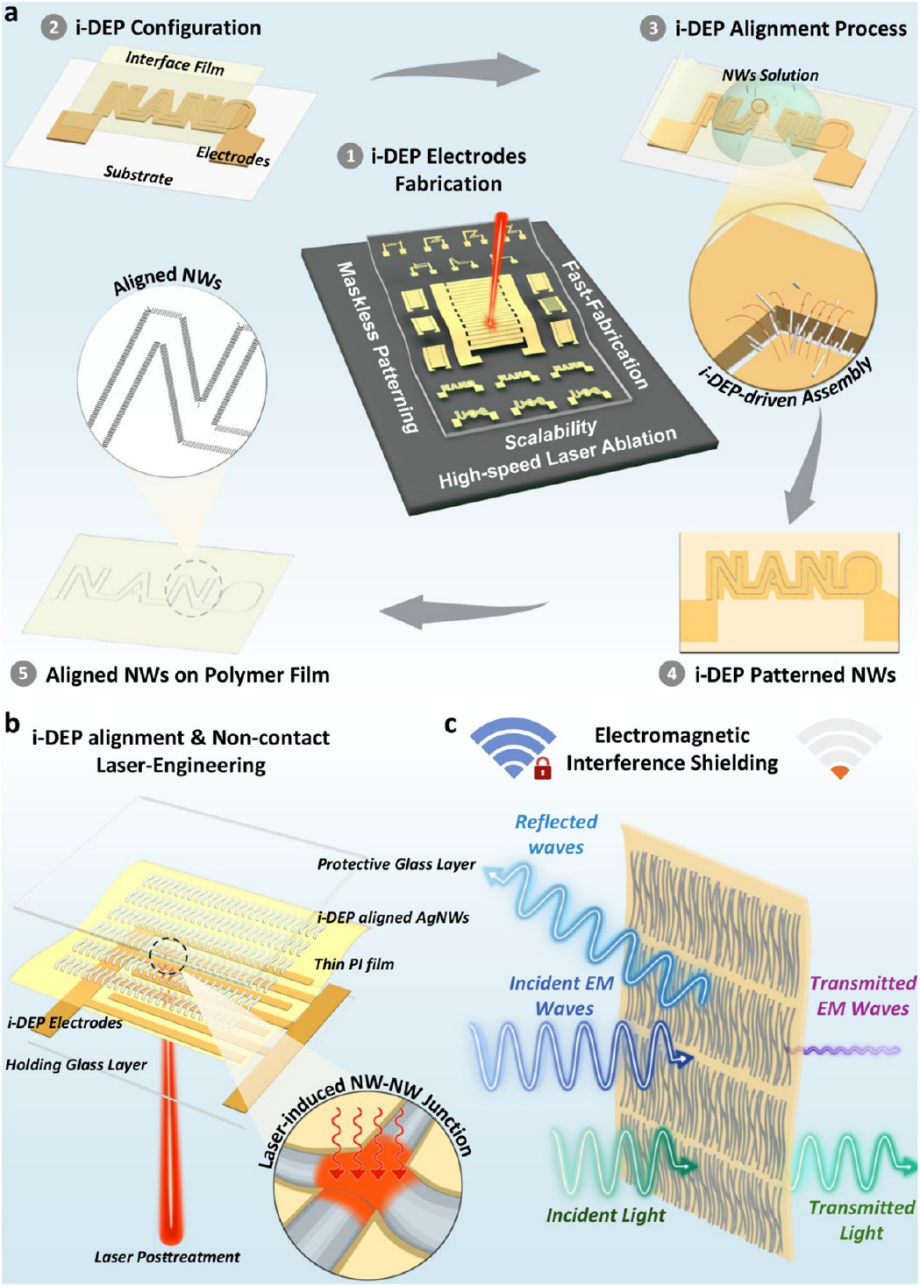
Conceptual illustration of the working principle of laser-engineered i-DEP aligned NW networks for flexible and transparent EMI shielding films. (a) High-speed, scalable picosecond laser ablation for direct fabrication of i-DEP electrodes (number 1), followed by i-DEP alignment of NWs on flexible polymeric thin films (Numbers 2–5), enabling precise translational and rotational assembly of NWs in a “NANO” pattern. (b) i-DEP alignment of large-area AgNW/PI thin films, combined with noncontact laser engineering for tailoring their performance through the photothermal effect, inducing solid NW–NW junction formation. (c) The laser-engineered i-DEP alignment approach yields highly flexible, optically transparent AgNW/PI composite EMI shielding films, indicating both high transmittance and strong shielding performance against unwanted EMI radiation.

## Results and Discussion

Surface morphology of the synthesized AgNWs was examined using a Field Emission Scanning Electron Microscope (FESEM) and Transmission Electron Microscope (TEM). FESEM images of AgNWs confirm the formation of AgNWs with a minimal number of nanoparticles observed (Figure 2a,b). Statistical analysis of the SEM images indicates that the AgNWs possess an average length of 31.4 ± 0.6 μm and an average diameter of 85.1 ± 0.6 nm, determined through Gaussian curve fitting, as shown in the inset of Figure 2a and b, respectively. These dimensions reveal a high aspect ratio (∼500) of NWs, reflecting their elongated morphology, which is further verified by the low-magnification TEM image in Figure 2c. Specifically, detailed diameter measurements were taken at multiple positions along their entire length, with the NWs exhibiting an average diameter of ∼39 nm (Figure 2d). A thin (∼2 nm) surface layer surrounding the AgNW is also observed, attributed to residual polyvinylpyrrolidone (PVP) from the synthesis process. High-resolution TEM imaging shows well-defined lattice fringes, presenting the crystalline nature of the AgNWs (Figure 2e). Specifically, the spacing between the lattice fringes was measured to be approximately 0.2 nm, assigned to the <111> plane. The selected area electron diffraction (SAED) pattern provides additional evidence of the high crystallinity of the synthesized AgNWs (Figure 2f). Additionally, the crystalline structure of the synthesized NWs was characterized by XRD analysis. A prominent peak at 38.93° corresponds to the (111) crystal plane, and other peaks at 45.2°, 64.89°, and 78.2° are attributed to the (200), (220), and (311) planes, respectively.^22^ These results demonstrate that the synthesized AgNWs exhibit a face-centered-cubic (FCC) crystal structure with a strong (111) diffraction peak, indicating a preferred orientation along the (111) planes. Furthermore, Raman spectroscopy was used to evaluate the structural characteristics of the AgNWs. The spectrum (Figure 2h) displays distinct vibrational modes at 230, 679, 1375, 1591, and 2931 cm^–1^. The sharp peak at 230 cm^–1^ is associated with Ag–O bonding vibrations (stretching mode).^23^ The vibrational peaks seen at 679 nm are attributed to reference C–S stretching vibration. Peaks at 1375 cm^–1^ and 1591 cm^–1^ arise from C=O and C–N vibrations, respectively, indicating interaction between PVP molecules and the NWs surface primarily through the oxygen atoms of C=O groups.^23^ The sharp peak at 2931 cm^–1^ corresponds to CH_2_ asymmetric stretching, reflecting the close association of the main chain of PVP ligands with the AgNWs surface.^24^ XPS analysis, confirming the presence of Ag, O, N, and C, is shown in the survey spectra in Figure S2a. The Ag 3d narrowband spectrum in Figure 2i illustrates doublets at 374.2 and 368.2 eV, corresponding to Ag 3d_3/2_ and Ag 3d_5/2_, respectively.^25,26^

**Figure 2:**
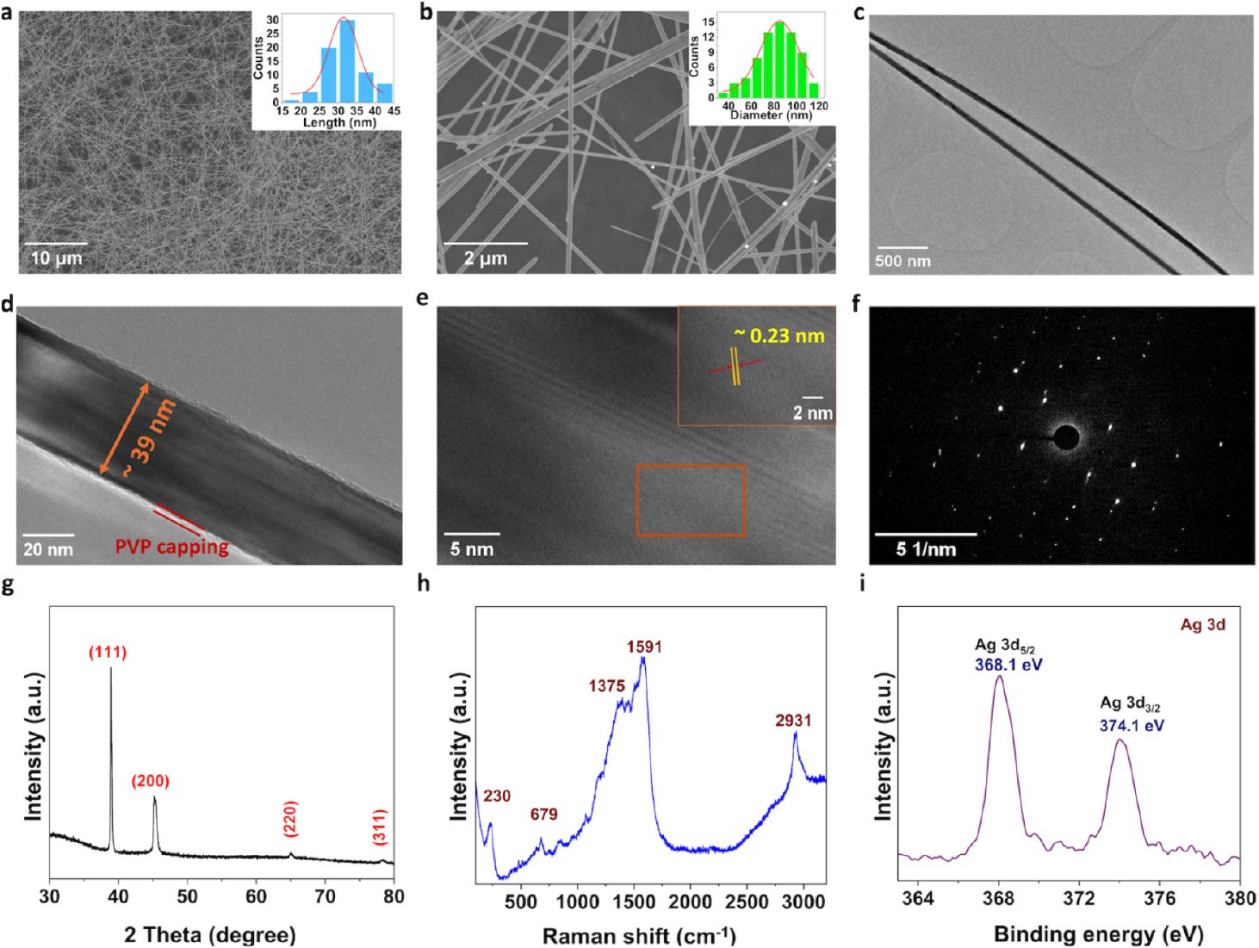
FESEM images of the synthesized AgNWs. (a) Demonstrating a high-density NWs with uniform dispersion across the substrate. (b) A magnified view showing long and ultrathin NWs, with insets presenting the length and diameter of NWs. (c–d) TEM images at low and high magnification demonstrate the structural integrity of the NWs, with an average diameter of ∼39 nm, and confirm the presence of a 2–5 nm PVP surfactant layer on the surface. (e) TEM image reveals clear lattice fringes indicating the crystalline nature of the AgNWs with a lattice spacing of 0.23 nm corresponding to the (111) crystal plane. (f) SAED pattern shows well-defined diffraction spots in a ring-like symmetry. (g) XRD pattern of AgNWs displays prominent spectral peaks corresponding to crystal planes (111), (200), (220), and (311). (h–i) RAMAN and XPS spectroscopy studies on the AgNWs demonstrate characteristic vibrational modes and a high-resolution narrow-band XPS spectrum of Ag 3d, respectively.

Precise spatial alignment of NWs on ultrathin and flexible substrates, with full control, high degrees of freedom, and high throughput, is crucial for manufacturing high-performance NW-based flexible electronic devices.^27,28^ Here, an interfacial DEP (i-DEP) strategy is introduced (Figure 3a), in which a flexible and transparent PI substrate is placed over the electrodes, acting as both an insulating barrier and the target substrate for indirect NW alignment on the interface through capacitive coupling.^29^ This configuration eliminates the need for intermediate layers and NW transfer steps, enabling direct and seamless NW assembly on the target substrate. It also provides effective isolation between the aligned NWs and the electrodes, enabling electrode sustainability by minimizing electrical damage.

**Figure 3:**
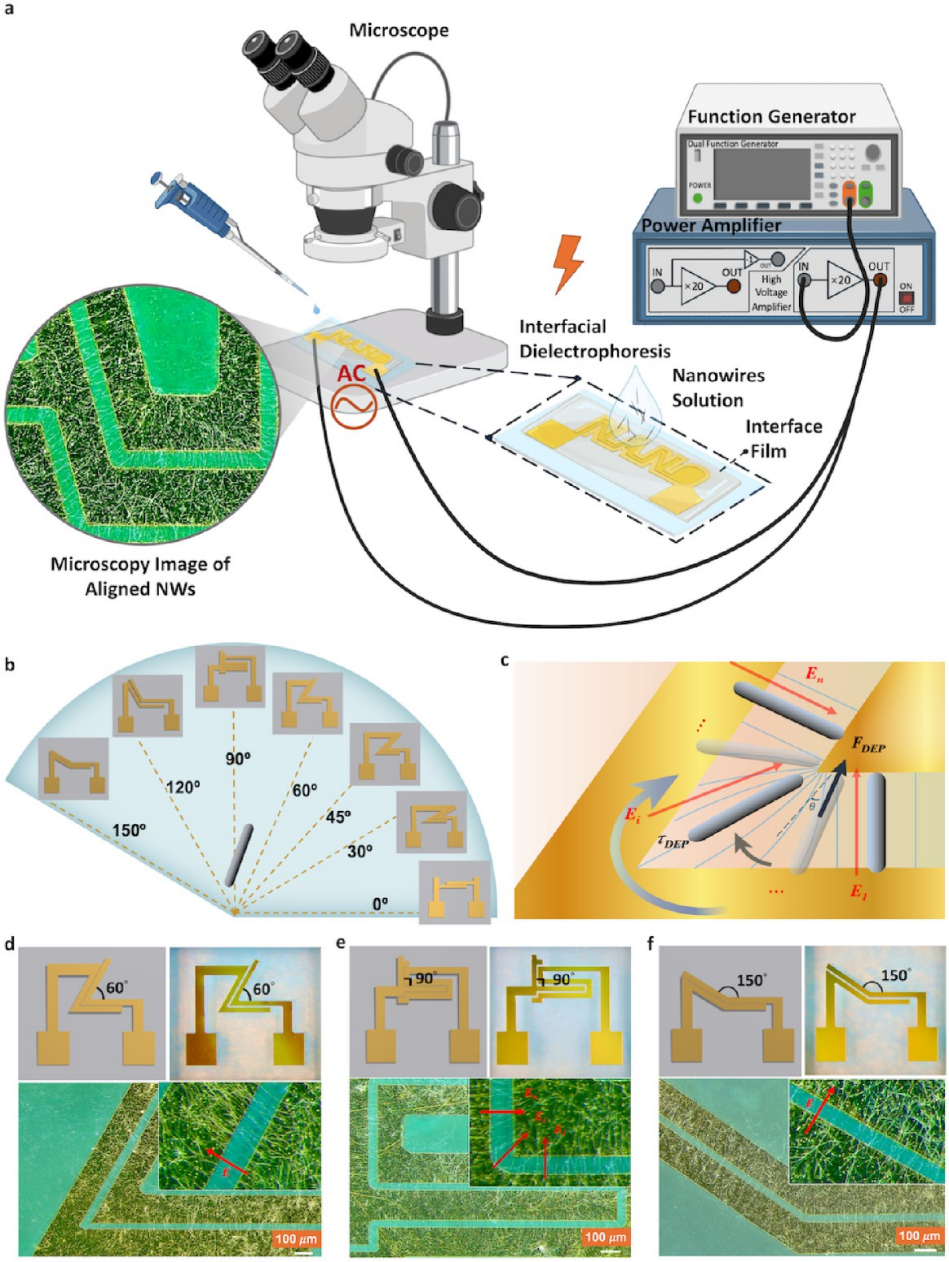
Schematic of the i-DEP assembly process and demonstration of high-precision angular alignment of NWs on PI films. (a) i-DEP experimental setup incorporating a signal generator and power amplifier, with the alignment process monitored by a high-resolution optical microscope; created in BioRender. Yalagala (2025) https://BioRender.com/a1878vy. (b) Laser-ablated angular electrodes ranging from 0° to 150° for directional NW alignment. (c) Schematic illustration of DEP-induced forces and torques acting on AgNWs between angularly arranged electrodes. (d–f) Schematics and corresponding prototypes of angular electrode pairs showing electric field directions during the positive half-cycle of the applied AC signal, along with the resulting precise i-DEP alignment of AgNWs at 60°, 90°, and 150°, respectively.

Using the proposed i-DEP method, the synthesized AgNWs were selectively aligned within the gaps (30 μm) between the electrode fingers by applying an AC voltage of 20 V_pp_ at 50 kHz (Figure 3). The precise assembly of NWs on ultrathin PI (5 μm) interfaces is driven by DEP forces and torques, which dominate over competing effects such as hydrodynamic drag and electrothermal forces.^30^ The DEP force acting on a NW in a nonuniform AC electric field can be expressed as^31^

1 $$F_{\mathrm{DEP}}=V\varepsilon_{m}Re\left(K\left(w\right)\right)\nabla \left({|E|}^{2}\right)$$

where *V* is a geometric-dependent factor (related to the NW volume), ∇(|*E*|^2^) denotes the gradient of the squared electric field magnitude, and *K*(*w*) is the Clausius–Mossotti (CM) factor defined as^32^

2 $$K\left(w\right)=\frac{\varepsilon_{p}^{*}-\varepsilon_{m}^{*}}{\varepsilon_{p}^{*}+L_{i}\left(\varepsilon_{p}^{*}-\varepsilon_{m}^{*}\right)}$$

3 $$\varepsilon^{*}=\varepsilon -j\frac{\sigma }{\omega }$$

Here, *ε* and *σ* denote the permittivity and conductivity of the NWs or medium, *L*_i_ is the depolarization factor along the NW’s longitudinal axis (*L*_∥_) or transverse axes (*L*_⊥_), and Re(K(w)) determines whether the NW is attracted ( Re(K(w))>0 , positive DEP) or repelled ( Re(K(w))<0 , negative DEP) from high electric field regions.

In addition to the translational force, anisotropic NWs experience a DEP-induced torque arising from the difference in polarizability (*α*_∥_) or (*α*_⊥_) along their axes, expressed as^33^

4 $$Im\left(\alpha_{∥}-\alpha_{⊥}\right)\propto Im\;(K_{∥}\left(w\right)-K_{⊥}\left(w\right))$$

5 $$\tau_{\mathrm{DEP}}=V\varepsilon_{m}Im\;(K_{∥}\left(w\right)-K_{⊥}\left(w\right)){|E|}^{2}\sin\left(2\theta \right)$$

where *θ* represents the angle between the NW axis and the electric field direction, and *K*_∥_(*w*) and *K*_⊥_(*w*) denote the CM factors along the longitudinal and transverse axes of the NW during the DEP process. The torque drives dynamic NW rotation, ultimately aligning their long axes with local electric-field lines (Figure 3c). Angular electrode designs and prototypes are shown in Figures 3b–f and S3, together with the corresponding static electric-field orientations during the positive half-cycle of the applied AC signal. In addition, these microscopy images capture the progressive angular reorientation and relocation of AgNWs across electrode pairs that orient from 0° to 150°, respectively. Specifically, eqs 1 and 5 indicate that both DEP force and DEP torque are proportional to the electric field squared magnitude (∇|*E*|^2^) and therefore remain unaffected by instantaneous field-direction reversals when an AC signal is applied to the electrodes. For example, in Figure 3e, AgNWs initially positioned in a vertical configuration were smoothly reoriented through a continuous 90° rotation to a fully horizontal alignment, passing through different intermediate angles (e.g., 45°). To demonstrate the simultaneous translational and orientational manipulation capability of the proposed i-DEP approach, two complex multiangle, location-specific electrode architectures, “NANO” and “UOG”, were designed and fabricated using a picosecond laser (Figure 4). In planar electrode systems, the DEP force is strongly influenced by the gradient of the squared electric field magnitude (∇|*E*|^2^), with the vertical (*z*-direction) component of this gradient often dominating the particle motion $\left(F_{DEP}\left(z\right)\propto \nabla \left|E\right|²\propto \frac{1}{Z^{3}}\right)$ .^34^ This effect is particularly significant when considering forces derived from the CM relation, which governs the particle polarizability and underlies classical DEP theory. Meanwhile, DEP-induced torque reorients the NWs by rotating their longitudinal axes to align with the field lines.^35^ The combined effect of strong DEP forces and torques steers the NWs to bridge electrode gaps with high spatial precision. This highlights the versatility and accuracy of the i-DEP method for scalable, reconfigurable, and rapid NW assembly on flexible interfaces, enabling seamless integration and manufacturing of high-performance NW-based electronics through an efficient and sustainable process.

**Figure 4:**
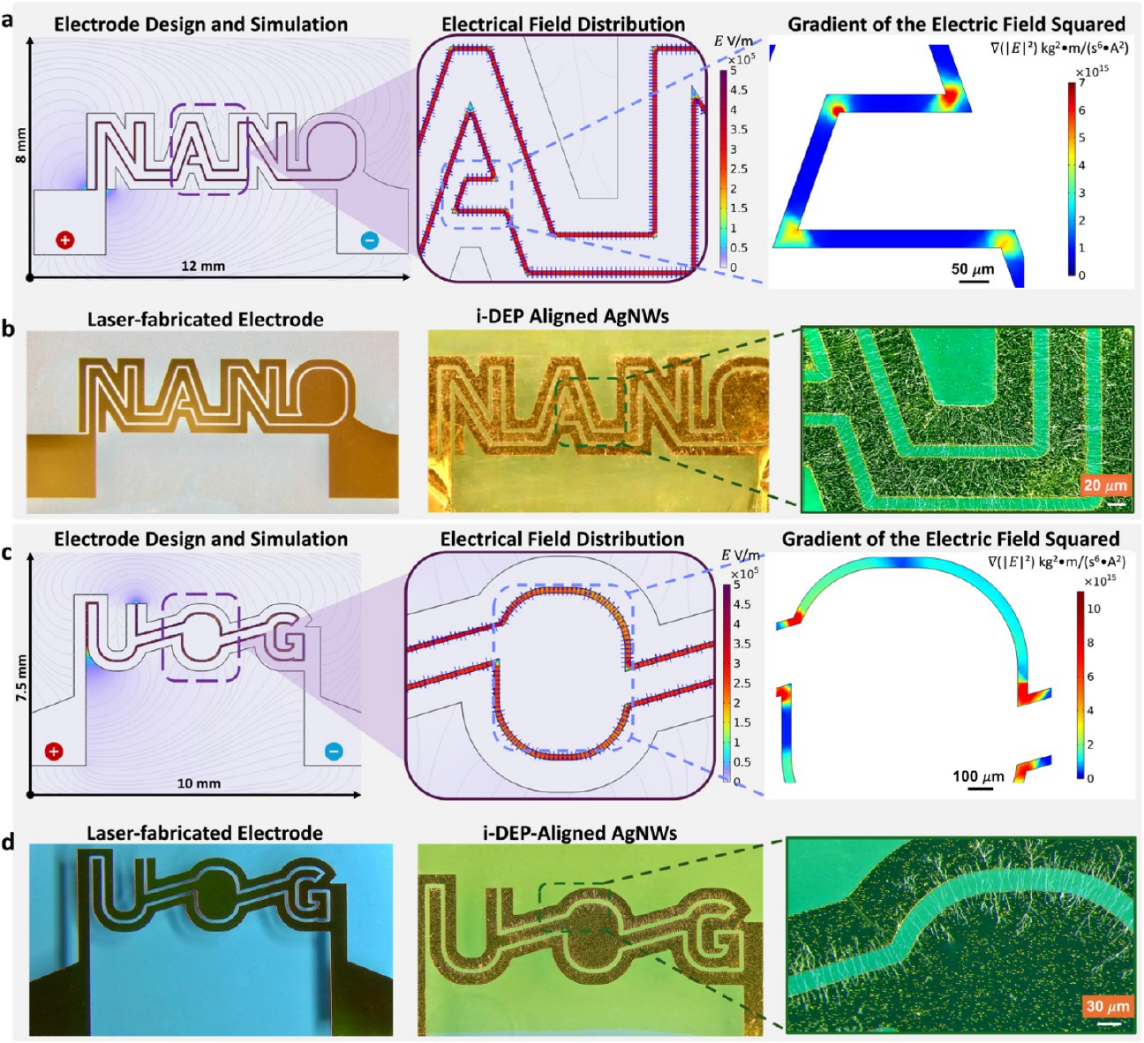
High-precision, full-control i-DEP alignment of AgNWs in multipath patterns on ultrathin PI substrates. (a) Simulated normalized electric field and electric field gradient squared (∇|E^2^ |) for the “NANO” electrode geometry. (b) Laser-ablated “NANO” electrodes enabling multidirectional AgNW assembly via i-DEP, demonstrating programmable NW “spelling”. (c) Simulated normalized electric field and ∇|E^2^ | for the “UOG” electrode pattern. (d) Experimentally AgNW assembly using the “UOG” laser-ablated electrodes, showing full angular orientation and programmable spatial positioning through the i-DEP method.

The high aspect ratio and high electrical conductivity (∼10^7^ S/m) of AgNWs make them a promising alternative to brittle transparent conductive oxides (e.g., indium tin oxide, ∼10^4^ S/m) for the fabrication of flexible and transparent EMI shielding films.^6^ Specifically, the electrical conductivity (*σ*) of the selected NWs is a critical parameter that governs the CM factor and the magnitude of the DEP force, as described in eqs 1–3. Typically, AgNWs-based EMI shielding films are fabricated via spray coating or spin-coating,^36^ where Rs is reduced by increasing NWs density or employing multilayer deposition.^37^ Additionally, the NW–NW junctions within these network are often weakly bonded, leading to high junction resistance that contributes to an overall large Rs over the AgNW network.^38,39^ Furthermore, dense, randomly oriented, multilayered AgNW networks sacrifice optical transmittance due to excessive overlapping and reduced void fraction.^40^ To overcome these limitations, a noncontact picosecond laser post-treatment is proposed to enhance both the electrical and optical properties of the AgNW networks while minimizing thermal distortion. Figure 5a schematically illustrates the i-DEP process for aligning a thin AgNW network on PI films using predefined interdigitated electrodes (IDEs). Following alignment, a noncontact laser post-treatment was performed on the AgNW/PI films, where the films were sandwiched between two thick, transparent glass slides and flipped so that the AgNW layer faced downward (Figures 5b–d and S4). This configuration provides a nanowelding strategy in which laser pulses traverse the glass slides without directly irradiating the NWs. The glass slide acts as a thermal buffer, dissipating excess heat and minimizing the thermal distortion of the NWs. Laser powers of 10, 20, 30, and 40 mW representing low, medium, high, and overtreatment levels were applied at a constant scanning speed of 670 mm/s (Table SI). SEM images of pristine and laser-treated AgNW networks (Figures 5e–g and S5a–f) reveal progressive NW junction welding as laser power increases from 10 to 30 mW, compared to the untreated network. Furthermore, AFM characterization (Figure S5g) further confirms the laser-induced nanowelding at the NW–NW junction sites. Quantitative analysis (Figure 5f) shows that the junction count increases from 29 to 58 within a confined area of the AgNW/PI film as the laser power rises. These NW–NW junctions are fused at targeted contact points due to localized electric field enhancement and confined photothermal effects from ultrafast laser pulses, resulting in low-defect NWs welding.^41^ At 40 mW, redundant energy input causes NWs breakage, marking the threshold for overtreatment (Figure S5f). During treatment, the high energy UV laser activates a strong surface-active state between the PVP and (100) crystal planes of AgNWs,^10,41^ facilitating localized interconnection and forming nanowelding at NW–NW junctions.^23^ The nanojoining yields efficient electron transport through stronger and more continuous conductive pathways within the AgNW network with minimal interfacial barriers, leading to a significant reduction in Rs from 1682 to 37 W/sq and a corresponding increase in electrical conductivity (Figure 5h). Meanwhile, Figures 5i,j and S6 compare the transmittance (% T) of bare PI, i-DEP aligned AgNWs/PI films, and i-DEP aligned AgNWs/PI films treated with varying laser powers. At 550 nm, transmittance improves from 73.9% to 83.1% after high-power laser welding, approaching the 88.7% transmittance of the bare PI substrate. TEM analysis (Figure 2d) reveals a thin (2–5 nm) layer of organic PVP surfactant residue on the surface of untreated AgNWs, which can absorb, scatter, or reflect incident light. Additionally, the untreated NW network contains weak interfacial contacts and discontinuous junctions that act as optical scattering sites. During laser treatment, localized heat facilitates the gradual decomposition and removal of excess PVP coating as the laser power increases from low to high levels. The melting of the PVP layers produces a cleaner NW network surface with reduced reflections and minimized light scattering at the film–air interface,^42^ resulting in a slight improvement in transmittance (Δ%*T* < 5%). Furthermore, laser-induced thermal diffusion enhances NW fusion, strengthening inter-NW connectivity and reducing structural irregularities.^23^ As a result, light scattering and optical haze are effectively suppressed, consistent with previously reported findings.^43–46^ Although a trade-off exists between Rs and %T, the optimized noncontact laser treatment method enables simultaneous enhancement of both by improving interfacial NWs quality rather than changing network density or employing additional layers, effectively removing the insulating PVP layers and inducing junction nanowelding. Subsequently, mechanical stability was further evaluated through current–voltage (*I*–*V*) measurements of untreated and laser-treated i-DEP aligned AgNW films under curvature angles from 30° to 180° (Figures 5i–k and S7). The laser-treated films exhibited superior mechanical flexibility and electrical robustness, showing resistance variation below ∼12% up to 120° due to solid welded nanojunctions and improved AgNW-PI adhesion (Figure S8). Overall, the proposed ultrafast laser post-treatment of i-DEP aligned AgNW/PI films provides an effective strategy for overcoming the inherent trade-off between electrical conductivity and optical transmittance. This approach holds strong potential for seamless adaptation to a broad range of transparent, 1D metallic NW-based flexible conductors and electrodes.

**Figure 5:**
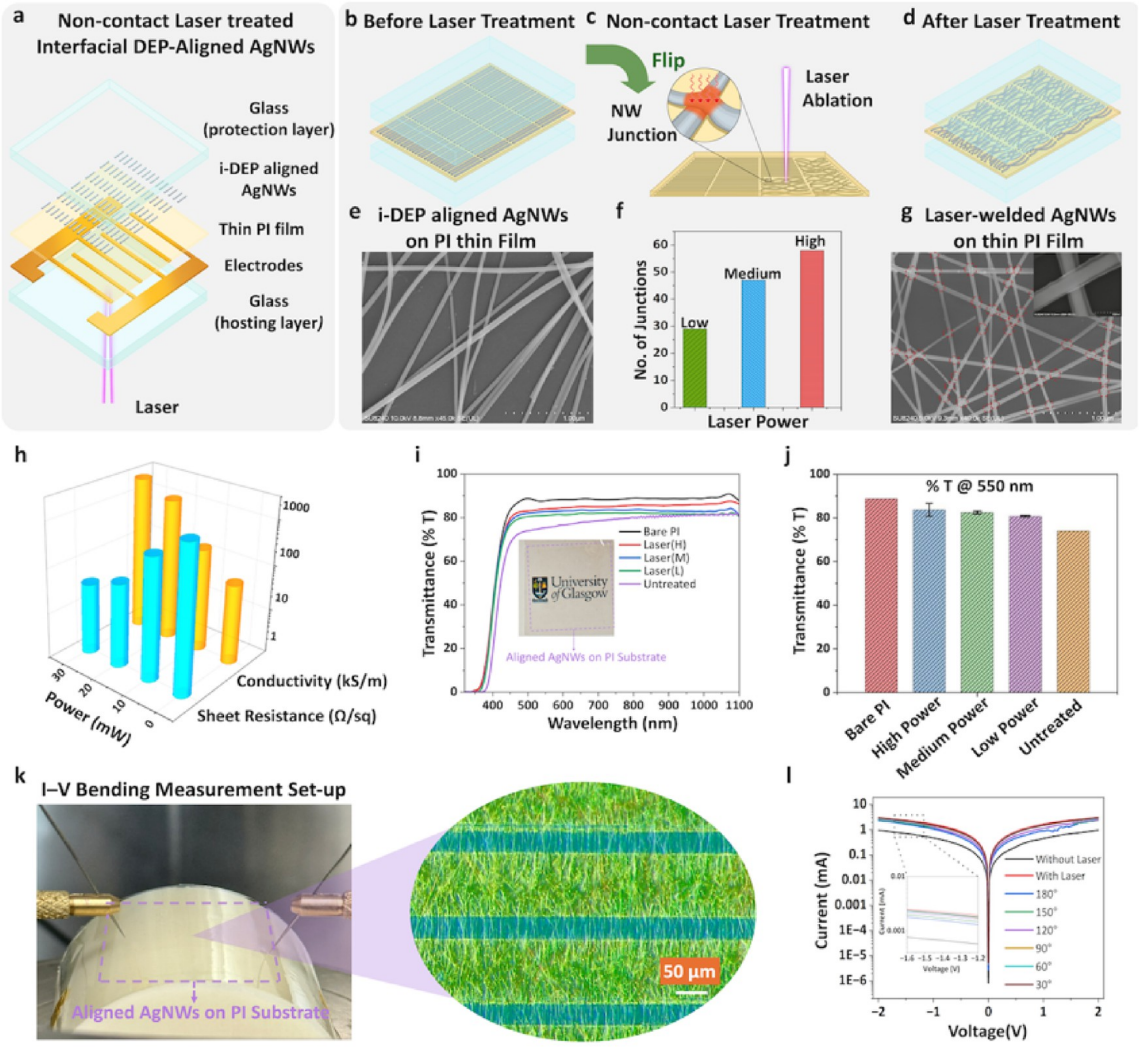
Fabrication, laser post-treatment, and performance evaluation of i-DEP aligned AgNW/PI films. (a) Schematic illustration of the i-DEP alignment process followed by noncontact picosecond laser post-treatment. (b, c) Detailed depiction of the laser treatment process. (e) SEM image of the i-DEP aligned AgNW/PI film before laser treatment. (f) Quantification of welded NW–NW junctions within a defined area under low (10 mW), medium (20 mW), and high (30 mW) laser power conditions. (g) SEM image of the AgNW network after laser treatment with a magnified view of a solid-fused NW–NW junction. (h) Comparison of Rs and electrical conductivity for untreated samples and those treated at different laser power levels. (i, j) Optical transmittance spectra and transmittance at 550 nm for bare PI, untreated i-DEP aligned films, and films subjected to varying laser powers; logo used with permission from https://www.gla.ac.uk/myglasgow/staff/brandtoolkit/brandelements/logo/. (k) *I*–*V* measurement setup under controlled bending on a probe station, with a magnified view of the i-DEP aligned AgNW/PI sample. (l) *I*–*V* curves of untreated and laser-treated films under different curvature angles (30–180°).

The outstanding level of control enabled by the laser post-treatment of i-DEP AgNW/PI films, combined with their enhanced conductivity, high optical transmittance, and robust mechanical stability, makes this approach a promising strategy for developing high-performance, transparent EMI shielding films. To investigate the EMI shielding potential of laser-treated i-DEP AgNW networks, two large-area films (40 cm^2^ and 80 cm^2^, inset of Figure 6a,b) were fabricated and measured across the 2.2–3.3 GHz and 3.3–6 GHz ranges, respectively. The overall 2.2–6 GHz broad frequency band covers widely utilized bands relevant to wireless communications, including Wi-Fi and radar systems.^21^ The EMI shielding measurements were performed on drop-cast, i-DEP-aligned, and i-DEP-aligned + laser-welded AgNW/PI films. Figure S9 depicts the schematic and optical images of the experimental setup. The reflection coefficient (*S*_11_) and transmission coefficient (*S*_21_) of EM waves across the two frequency ranges are presented in Figure S10. The EMI shielding effectiveness (SE), shown in Figure 6a,b, was calculated using the following equation, which accounts for reflections from the substrate material:^21,47^

6 $$SE\left(dB\right)=S_{21}^{\mathrm{substrate}}-S_{21}^{\mathrm{meas}}$$

where $S_{21}^{\mathrm{substrate}}$ is the transmission coefficient of the EM signal through the pristine PI substrate, and $S_{21}^{\mathrm{meas}}$ is the transmission coefficient through the AgNW-coated film.

**Figure 6:**
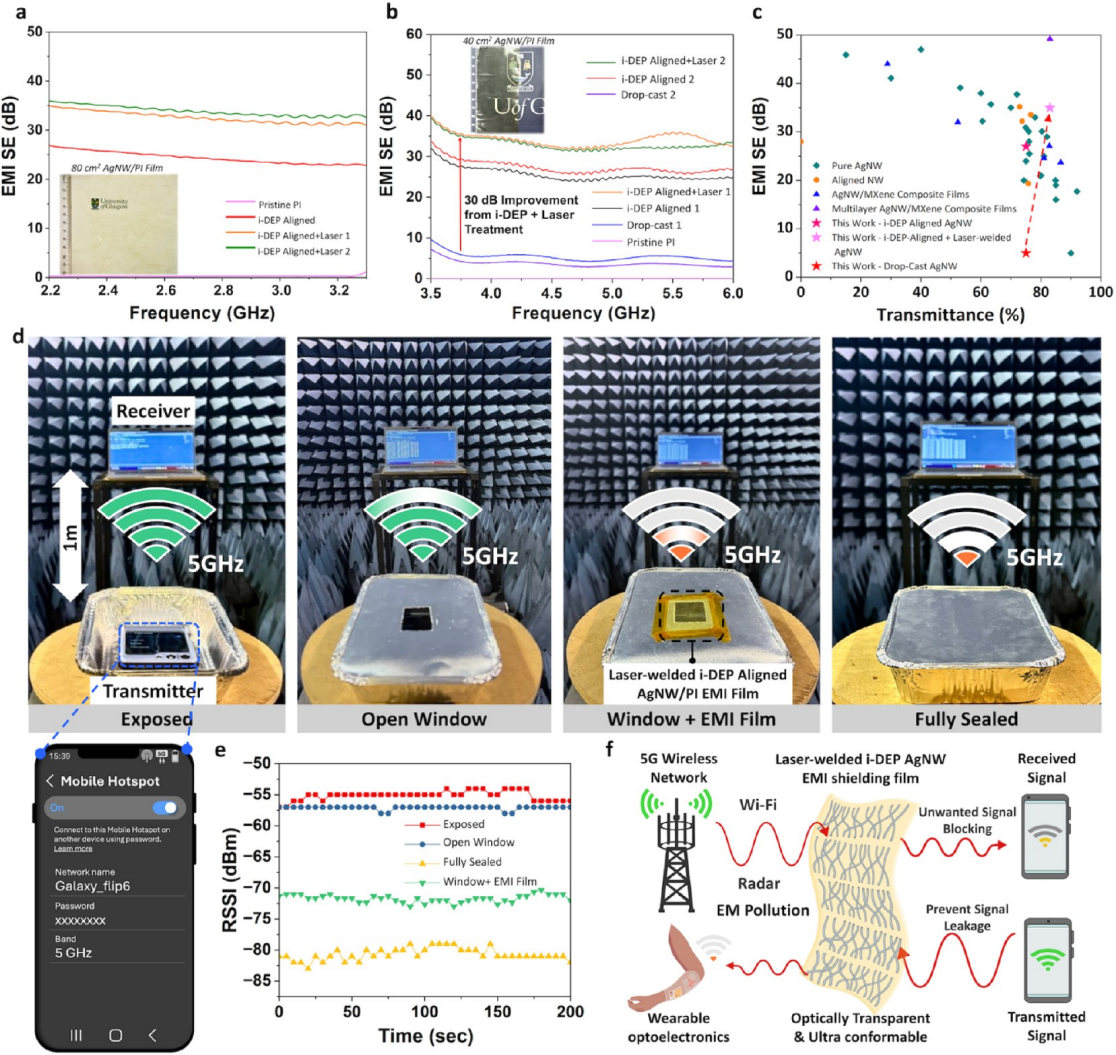
(a) Measured EMI SE of large-area (80 cm^2^) AgNW/PI thin films across the 2.2–3.3 GHz frequency range. (b) Measured EMI SE of 40 cm^2^ samples evaluated in the 3.3–6 GHz frequency range; logo used with permission from https://www.gla.ac.uk/myglasgow/staff/brandtoolkit/brandelements/logo/. (c) Comparison of EMI SE and optical transmittance of this work with other AgNW/polymer composite films reported in the literature. (d) Real-time RSSI measurement setup within an anechoic chamber under different test scenarios: exposed, open window, open window covered with laser-welded i-DEP aligned AgNW/PI film, and fully blocked. A mobile phone serves as a 5 GHz Wi-Fi signal transmitter. (e) Measured RSSI over 200 s for all test scenarios. (f) Illustration of the proposed high-performance EMI films for blocking multipath interference and complex EM pollution in flexible electronics and electronic skin display systems.

Pristine large-area drop-cast films (80 cm^2^) exhibit S_11_ ≈−5 dB (Figure S10) across 2.2–3.3 GHz, indicating weak conductive continuity. Meanwhile, the 40 cm^2^ drop-cast AgNW/PI films show comparable performance, providing 4.5 dB of EMI SE in the 3.5–6 GHz range, corresponding to ∼60.2% shielding efficiency. In comparison, the i-DEP-aligned films demonstrate a marked improvement in shielding performance, achieving to ∼25 and 27 dB SE for two large-area samples (Figure 6a,b), representing ∼99.7% and ∼99.8% shielding efficiency, respectively. The well-defined NW alignment (Figure 5k) introduces narrow gaps between adjacent nanowires that function as micro- and nanocapacitors, forming a capacitively coupled interwire network that enhances local electric-field coupling and displacement current flow. Such coupling effect, enabled by i-DEP-induced structural alignment, have been proved by previous studies to strongly influence EMI shielding mechanisms beyond contributions from bulk conductivity alone.^19,20^ When combined with laser welding, i-DEP-aligned AgNW/PI film (∼5.1 μm thickness) achieves ∼35 dB SE over large areas (40 and 80 cm²), effectively blocking more than 99.97% of incident EM radiation across the entire 2.2–6 GHz band. Remarkably, the experiment demonstrates that the i-DEP-aligned NWs can achieve more than 1000× (30 dB) improvement in shielding performance compared to randomly oriented drop-cast NWs. To further investigate the electrical properties of the laser-treated i-DEP-aligned AgNW films, complex impedance measurements were performed from 100 kHz to 1 GHz. As shown in Figure S11, the AgNW films exhibit a nearly constant real impedance, indicating that the overall complex impedance is dominated by resistive behavior. Following laser treatment, NW–NW junctions are fused, reducing interwire gaps and forming a predominantly ohmic network with enhanced electrical continuity. These results demonstrate that laser-welded i-DEP-aligned AgNW/PI films achieve superior EMI SE, primarily governed by ohmic reflection, with additional contributions that may arise from residual nanoscale gap coupling within the aligned structure.

This study further showcases the capability of an ultrathin, highly ordered AgNW/PI film to deliver robust and uniform EMI shielding at scalable dimensions. To evaluate the performance of laser-treated i-DEP-aligned AgNWs relative to state-of-the-art transparent and semitransparent NW-based shielding materials,^48–54^ Figure 6c and Table SII present a comprehensive comparison of SE and optical transparency across random, aligned, laser-treated, and i-DEP-aligned NW networks, along with representative shielding films including AgNW films, AgNW/dielectric polymer composites, and other aligned nanostructure films. Most reports reveal that there is a clear trade-off between optical transparency and SE; however, the laser-treated, i-DEP-aligned AgNW thin film achieves among the highest shielding efficiency while maintaining excellent transparency for single-layer configuration. Specifically, the reported multilayered structure, in which AgNWs are paired with Ti_3_C_2_T_*x*_ MXene coating, achieves enhanced EMI shielding efficiency up to 99.99%, but at the cost of an overall thickness approaching a centimeter.^56^

To assess the practical EMI shielding performance of the AgNW/PI films, real-time received signal strength indicator (RSSI) measurements were conducted at the widely used 5 GHz Wi-Fi band inside an anechoic chamber. As illustrated in Figure 6d,e, a mobile phone serving as a Wi-Fi hotspot transmitter (Tx) radiates an EM signal at 5 GHz, while a laptop running a custom program continuously records the RSSI over 200 s at a 1-m distance. The Tx was placed in a metal box under four test scenarios: open exposure, open window, open window covered with a laser-welded i-DEP-aligned AgNW/PI film, and fully metal-blocked. RSSI values for the exposure and open window conditions were −55 and −57 dBm, respectively, demonstrating strong EM signal reception. In contrast, the fully metal-blocked case exhibited the lowest RSSI at −80 dBm, proving a near-complete signal blockage. Notably, the fabricated EMI film covering the cutout window reduced the RSSI by ∼15 dBm, corresponding to roughly a 30-fold decrease in signal power, highlighting its effectiveness in blocking EM waves at the target frequency. It is worth noting that the lower shielding observed compared to waveguide measurements is due to the poor sealing of the metal box’s imperfect contact with the thin film-coated window. These results indicate the potential of the fabricated transparent and conductive EMI film to effectively block unwanted EM signals, ensuring reliable and uninterrupted operation of smart wireless electronics in complex EM environments (Figure 6f). Future efforts may focus on pairing laser-treated i-DEP-aligned AgNW networks with highly conductive top layers or multilayer composite structures to further advance the development of highly ordered, ultrathin, and transparent EMI shielding films.^55,56^

## Conclusion

In summary, we introduce a complete workflow that integrates rapid micro/nanoelectrode fabrication, precise i-DEP NW alignment, and targeted post-treatment of NW networks for property engineering toward the manufacturing of high-performance NW-based electronic devices. A simple, scalable, and cost-effective high-speed laser ablation method was demonstrated for the maskless fabrication of diverse design patterns. Alignment of synthesized AgNWs was demonstrated using the i-DEP strategy, offering full spatial control over NW orientation and placement and enabling the programmability of NWs into complex architectures. Subsequent laser-induced NW–NW junctions significantly reduced sheet resistance (∼46×) with slight improvement in optical transmittance (10%), addressing the long-standing trade-off between electrical and optical performance in transparent and conductive films. The ultrathin, large-area laser-treated i-DEP AgNW/PI film (∼5.1 μm) achieved ∼35 dB EMI shielding effectiveness across the 2.2–6 GHz frequency band, blocking over 99.97% of incident signals while maintaining 83.1% optical transmittance. The combination of the i-DEP assembly with customizable laser post-treatment enables controlled modification of material properties, providing a versatile and precise route for fabricating a broad range of high-performance NW-based flexible composite electronic devices.

## Experimental Methods

### Materials and Characterization

All of the chemicals used for the synthesis of AgNWs were purchased from Merck and were used as received. Raman spectral analysis was carried out using the LabRAM HR system with an excitation wavelength of 532 nm. XRD data were collected using a Rigaku XRD tool with a Cu Kα radiation source. X-ray Photoelectron Spectroscopy (XPS) analysis was performed using a Kratos AXIS Supra+. Nanometer-scale imaging was conducted with a JEOL ARM200CF-MAgTEM. To study surface morphology, FESEM analysis was carried out using a Hitachi SU8240. Samples were prepared by drop-casting ∼0.2 mg/mL of AgNW solution onto a cleaned ITO substrate. Sheet resistance was measured using a 4-point probe system (Ossila Ltd., T2001A5). Optical properties of AgNW/PI films were characterized with a PG Instruments T65 UV–Vis spectrophotometer at room temperature, and a Keysight B2912A source meter was used for electrical measurements.

### Synthesis of AgNWs

Here, a traditional polyol method was adopted to prepare the AgNWs. The polyol method was carried out by the reduction of silver oxides or silver salts with reducing agents, crystal seeds, and polyols. In a typical AgNWs synthesis, three solutions were prepared: solution 1 (S1): 500 mg of AgNO_3_ in 20 mL of ethylene glycol (EG); solution 2 (S2): 116.8 mg of sodium chloride (NaCl) in 20 mL of EG; solution 3 (S3): 400 mg of PVP (36000 MW) in 20 mL of EG. Later, 10 mL of EG, 2 mL of solution 2, and 10 mL of solution 1 and 3 are added into a 100 mL conical flask. The conical flask was then placed on a preheated hot plate at 160 °C for 2 h, with the mixture continuously stirred at 400 rpm. The mixture solution gradually turned opaque. The resulting solution was cooled to room temperature, and the synthesized AgNWs were characterized. The complete procedure is depicted in Figure S1.

### Fabrication of I-DEP Electrodes using a Picosecond Laser

A borosilicate glass slide was cleaned using methanol, 2-propanol, acetone, and deionized (DI) water, respectively, via the ultrasonication method and was used as a substrate for all the i-DEP electrodes. Next, a Ti/Au bilayer (10 nm Ti/200 nm Au) was deposited onto the glass slides as the electrode material for the fabrication of the i-DEP electrodes. A UV-based picosecond laser (LPKF ProtoLaser U4, 335 nm wavelength) with built-in software (LPKF CircuitPro PL) was used for electrode fabrication. Clean etching of the conductive films with minimal residual gold nanoparticles was achieved using optimized laser parameters: a hatch power of 1.28 W, scanning speed of 189 mm/s, frequency of 75 kHz, and four repetitions with different hatching angles. Etching was performed at strip lines with angles of 0°, 22.5°, 67.5°, and 90° relative to the *y*-axis. The fabricated i-DEP electrodes were then cleaned with isopropyl alcohol (IPA) to eliminate any residual materials. The electrodes were inspected under a microscope and tested with a multimeter to confirm their structural integrity and the absence of short circuits between parallel electrodes.

### i-DEP Alignment of AgNWs on Flexible Thin PI Film

An AC signal at 50 kHz with 1 V peak-to-peak, generated by a function generator (AIM-TTi TGF4242), was amplified 20× using a power amplifier (FLC Electronics A400DI) and applied to the electrode pads. A droplet of AgNW suspension was cast onto a thin polyimide (PI) film (5 μm) on top of the electrodes. NWs became polarized under the applied AC field and were rapidly attracted toward regions of high electric field gradient, aligning and bridging the electrode gaps according to the predefined pattern. A representative “NANO” pattern demonstrates this spatially controlled assembly, as shown in Figure 3. The i-DEP alignment process was monitored in real time using an optical microscope (LEICA M165 C), confirming the precise, multidirectional alignment of AgNWs along complex, contoured electrode geometries.

### Noncontact NW-Network Nanowelding using Picosecond Laser Post-Treatment

The proposed noncontact strategy (Figure S4b), which uses two transparent and thick glass slides, aims to absorb excess heat to protect the AgNWs and enable optimized laser melting of the PVP polymer coating on the surface of the AgNW, as observed from the TEM, SEM, and AFM images in Figures 1 and S5. Systematic optimization studies were performed to properly weld the NW–NW junction through the photothermal effect while minimizing thermal damage by varying different laser powers. Various parameters, including the laser power, scanning speed, frequency, and number of repetitions, along with the corresponding NW–NW junction numbers in a localized area, were studied and detailed in Table S1.

### Electromagnetic Interference (EMI) Shielding

EMI shielding measurements were taken using two pairs of rectangular waveguides, WR187 (47.54 mm × 22.15 mm) and WR340 (86.36 mm × 43.18 mm), connected to a Vector Network Analyzer (VNA) from Pico Technology (PicoVNA 106, covering 300 kHz–6 GHz) with phase-stable Mini-Circuits CBL-1.5M-SMSM+ cables (*L* = 1.5 m). The VNA was calibrated in the operating range of the waveguides (2.2–6 GHz) using a through calibration. AgNW samples deposited on polyimide were placed between the waveguides and sealed with clamps to prevent signal leakage.

## Supplementary Material



## Notes

Additional details on AgNW synthesis and characterization, laser parameters, experimental setups, and methodology, along with electrical parameters of i-DEP aligned AgNW/PI EMI films (PDF)
