# Supplementary material for: Laser-Engineered Interfacial Dielectrophoresis-Aligned Nanowire Networks for Transparent Electromagnetic Interference-Shielding Films
Source: ACS Nano. 2025 Dec 16;19(51):42760–71. doi: 10.1021/acsnano.5c13772 (PMC12756913; doi:10.1021/acsnano.5c13772)
Supplement: Supplementary file 1 [file nn5c13772_si_001.pdf]

Supporting Information

Laser-Engineered Interfacial-Dielectrophoresis Aligned Nanowire Networks for Transparent Electromagnetic Interference Shielding Films

Jungang Zhang[1], Venkatarao Selamneni[2], Bhavani Prasad Yalagala[1], Benjamin King[3], Jiaoran Wang[1], Luvsanbat Khurelbaatar[1], Carlos García Núñez[1], Mahmoud Wagih[3], Morteza Amjadi[2*], Hadi Heidari[1*]

[1]Microelectronics Laboratory (meLAB), James Watt School of Engineering, University of Glasgow, G12 8QQ Glasgow, U.K.
[2]Biomedical Soft Robotics Group, James Watt School of Engineering, University of Glasgow, G12 8QQ Glasgow, U.K.
[3]Green RF-Enabled Electronics Laboratory, James Watt School of Engineering, University of Glasgow, G12 8QQ Glasgow, U.K.
Corresponding author: Morteza Amjadi[2], morteza.amjadi@glasgow.ac.uk;

Hadi Heidari[1*], hadi.heidari@glasgow.ac.uk.

Table SI: Optimization Studies of Laser-treatment Parameters

Mode of treatment	Power (mW)	Speed (mm/s)	Repetition (times)	Frequency (kHz)	Pattern style/angle	NW-NW junction No.
**Low power**	10	650	1	30	Strip lines @ 22.5° to the y-axis	29
**Medium power**	20	650	1	30	Strip lines @ 22.5° to the y-axis	47

**High power**	30	650	1	30	Strip lines @ 22.5° to the y-axis	58
**Overtreated Power**	40	650	1	30	Strip lines @ 22.5° to the y-axis	NWs Damage

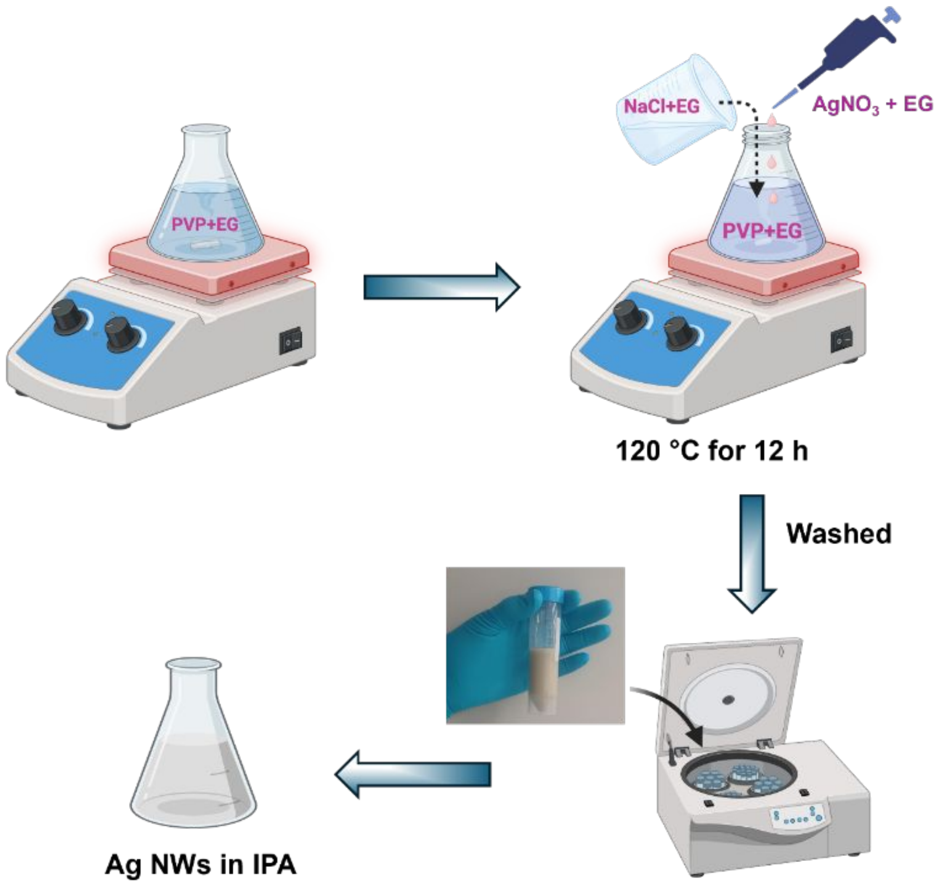

Figure S1: Schematic diagram of AgNWs synthesis using polyol method.

High resolution C 1s spectrum of synthesized AgNWs is shown Figure S2b and it reveals two prominent peaks at 284.6 eV and 288.3 eV. The significant peak at 284.6 eV represents C-H/C-C bonds and another peak at 288.3 eV is attributed to the O-C=O bonds originating from PVP residual during the preparation of Ag NWs.

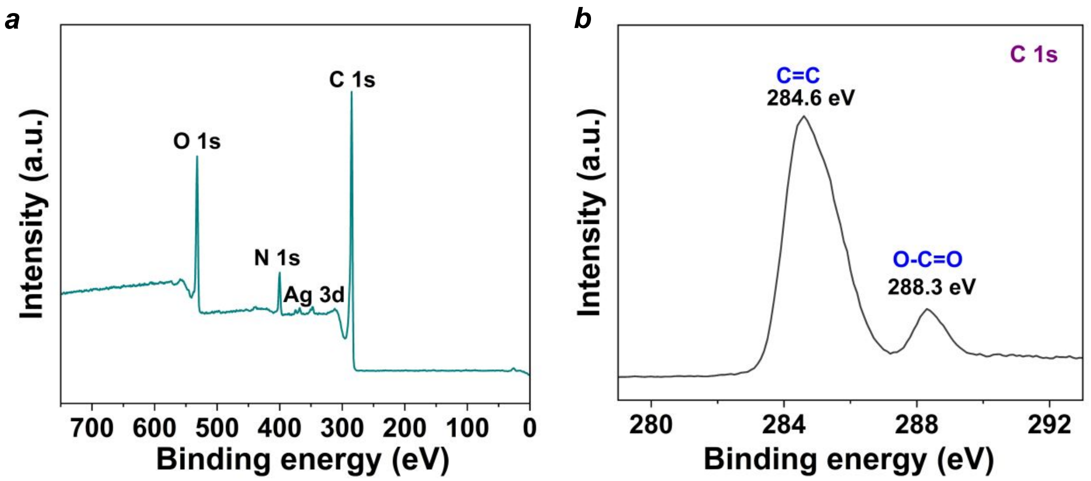

Figure S2. (a) Wide scan spectra. (b) Narrowband C 1s of synthesized Ag NWs.

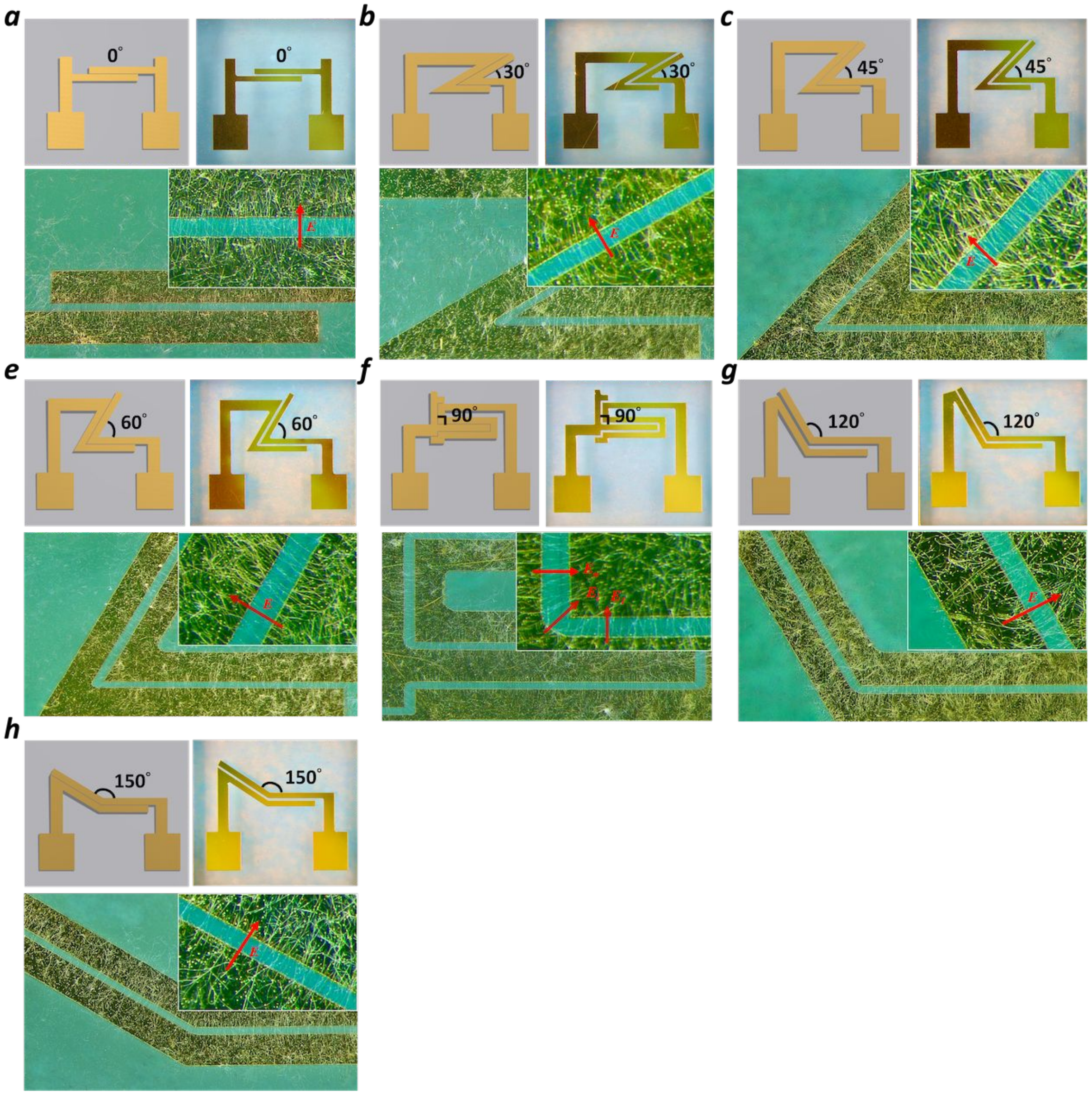

Figure S3. (a-h) Design and fabrication of individual angular electrodes with varying angles

from 0° to 150°, with corresponding rotational control of NWs using the i-DEP methods, demonstrating AgNWs alignment in the direction of the applied electric field.

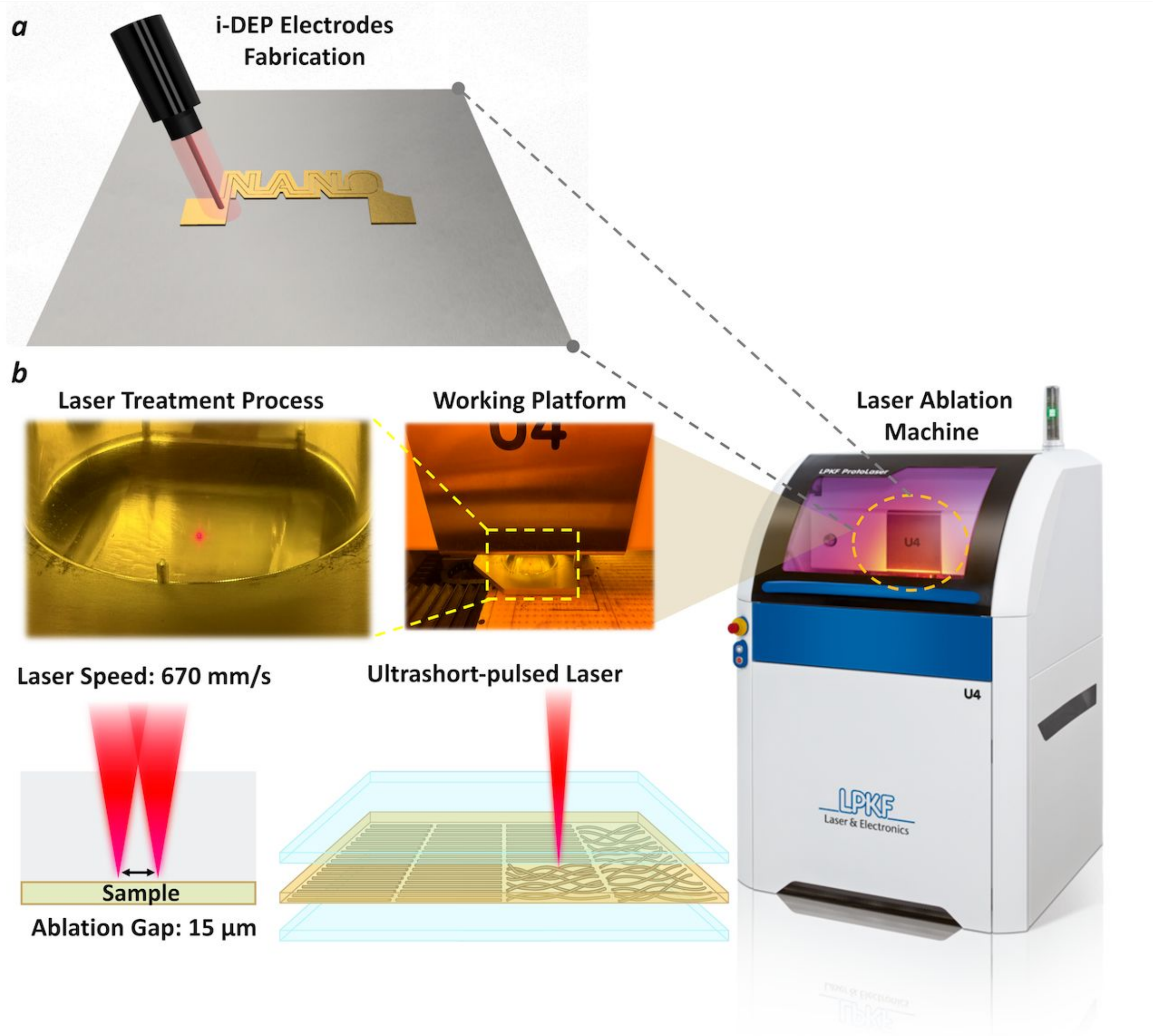

Figure S4: (a) Schematic representation of the fabrication process for i-DEP electrodes consisting of a Ti/Au later (10/200 nm) using ultra-fast laser patterning. (b) Process flow diagram and optical images illustrating the non-contact laser post-treatment for nanowelding of AgNW/PI films. The treatment was performed at a laser scanning speed of 670 mm/s with an ablation strip gap of 15 µm. Two transparent glass slides were placed on the top and bottom of the AgNW/PI film to protect from excessive damage during the laser-treatment.

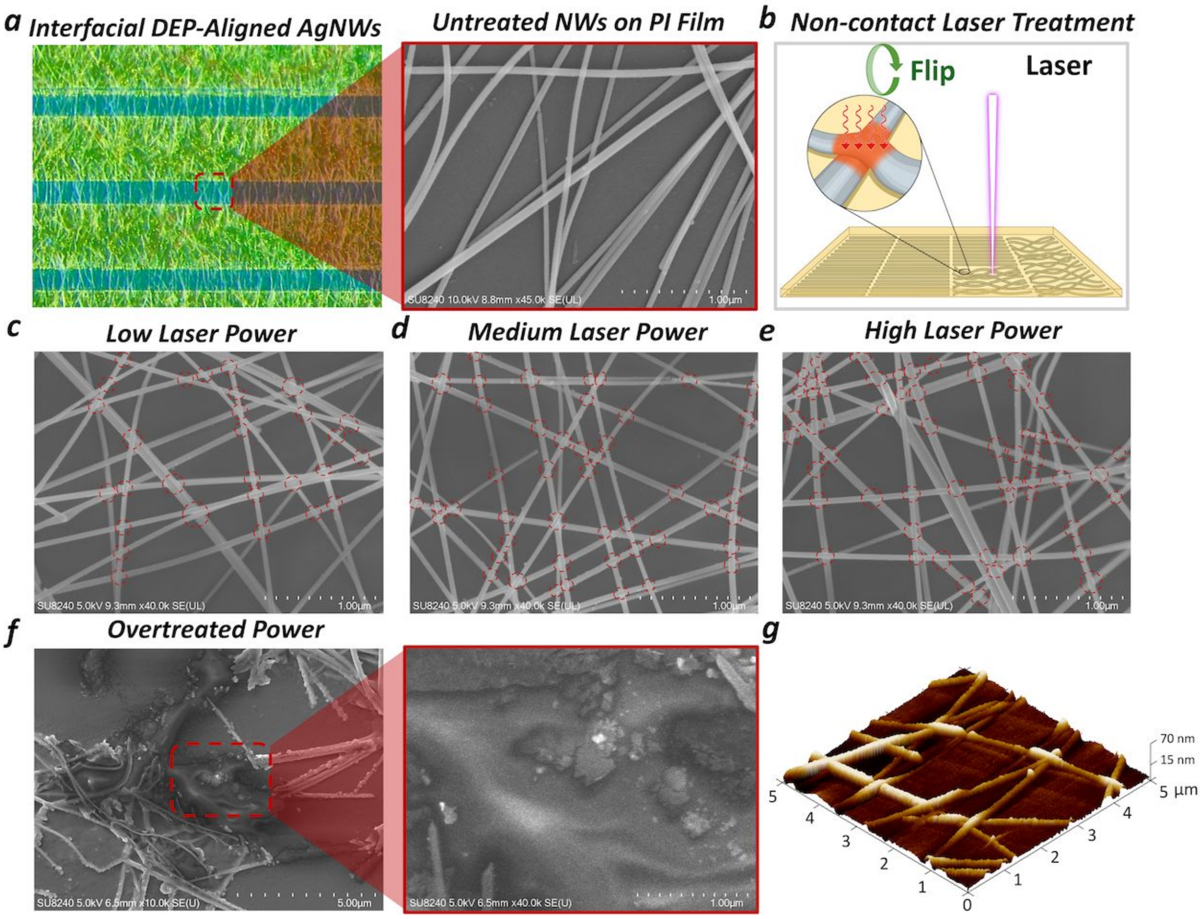

Figure S5. (a) i-DEP aligned silver nanowires on IDE patterned electrodes with a gap of 30 µm with a magnified SEM image of the aligned AgNWs is shown on the right. (b) Schematic illustration of laser treatment-based NWs nanowelding process. (c)-(e) SEM images of the nanowelded AgNWs subjected to laser powers of 10, 20 and 30 mW, respectively. (f) SEM image showing AgNWs thermal damage caused by excessive laser power (> 30 mW), with a zoomed in the image illustrating the rupture and distortion of AgNWs, (g) AFM topography image showing nanowire–nanowire (NW–NW) interconnections in laser-treated Ag nanowires (AgNWs) deposited on a transparent and flexible polyimide (PI) film (thickness: 5 µm).

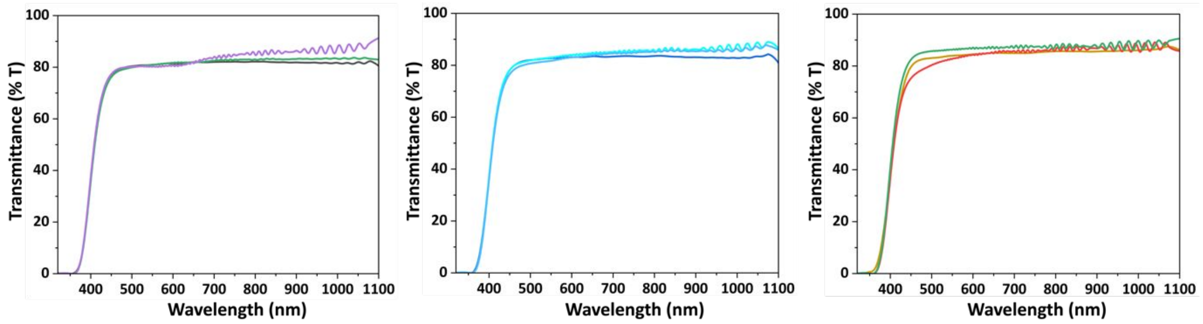

Figure S6. (a)-(c) Transmittance measurements at different laser power levels, corresponding to low, medium, and high laser power treatments, respectively.

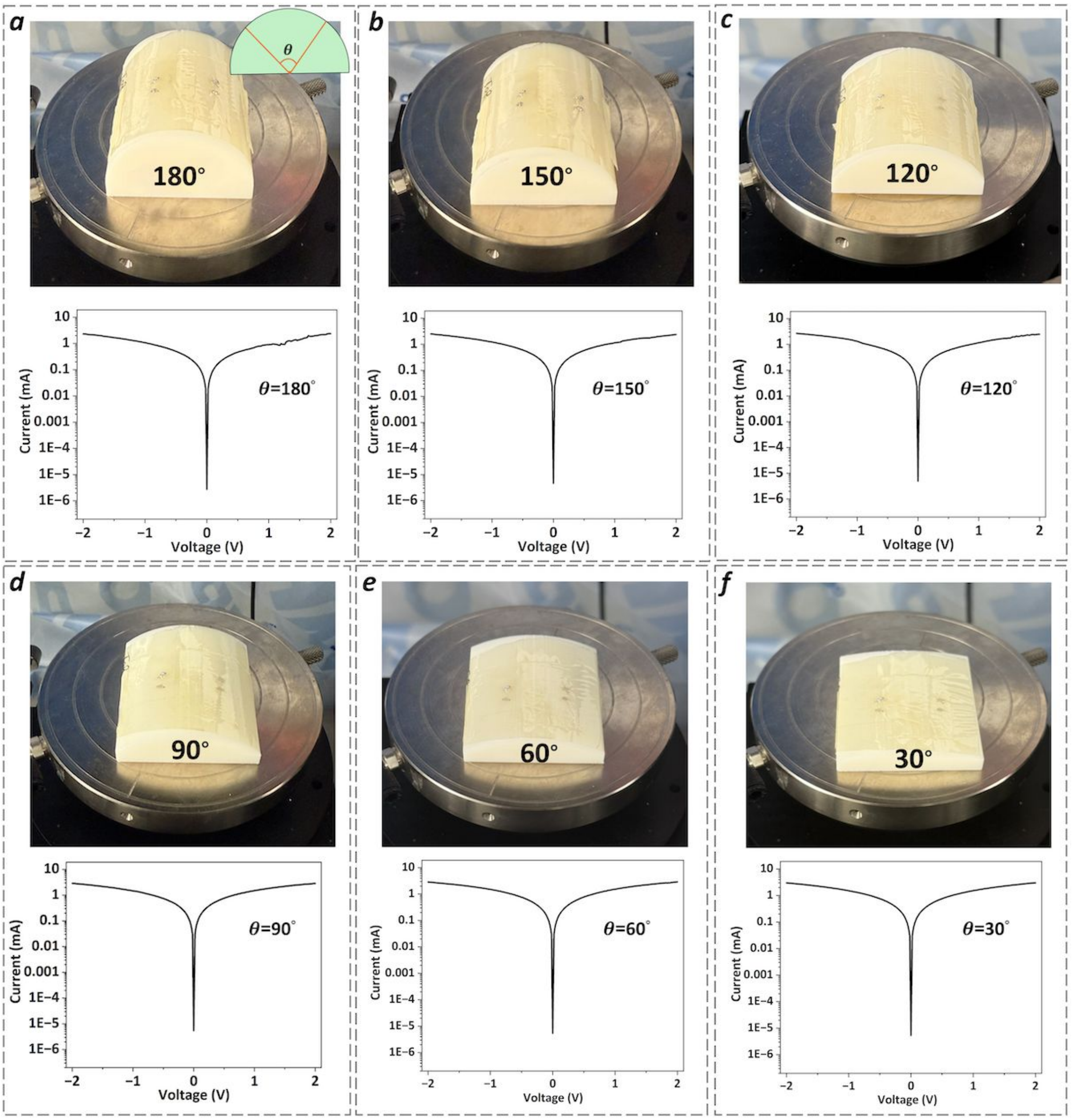

Figure S7. (a-f) Mechanical bending tests performed on the laser-treated, i-DEP aligned AgNWs/PI film under varying bending angles ranging from 30°-180°, using 3D-printed models with different bending radius.

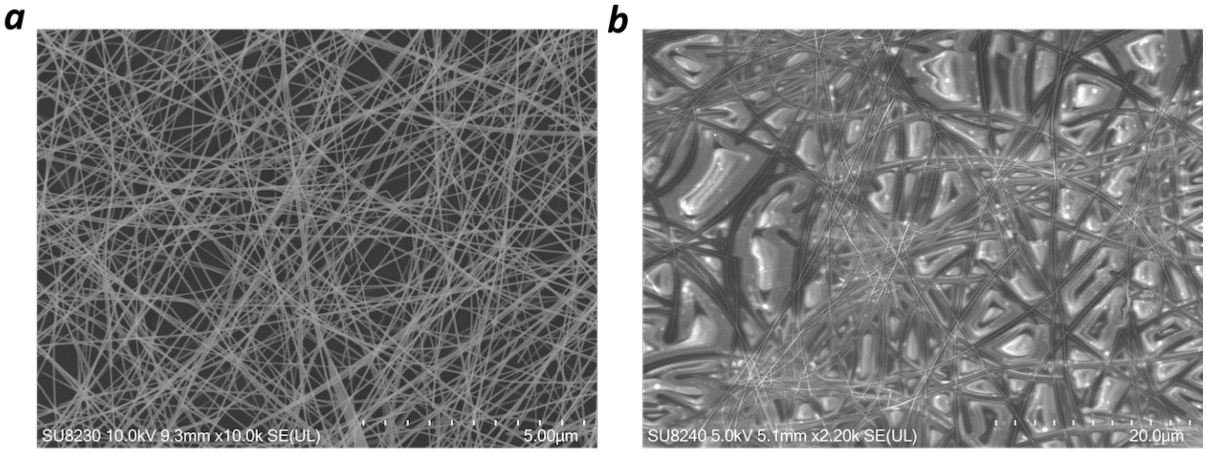

Figure S8. SEM analysis of AgNW/PI film surface adhesion. (a) Before the laser treatment, showing no interaction between the AgNWs and the PI surface. (b) after the laser treatment with a high power of 30 mW, demonstrating strong adhesion between the AgNWs and the PI substrate, attributed to increased heat absorption and localized melting.

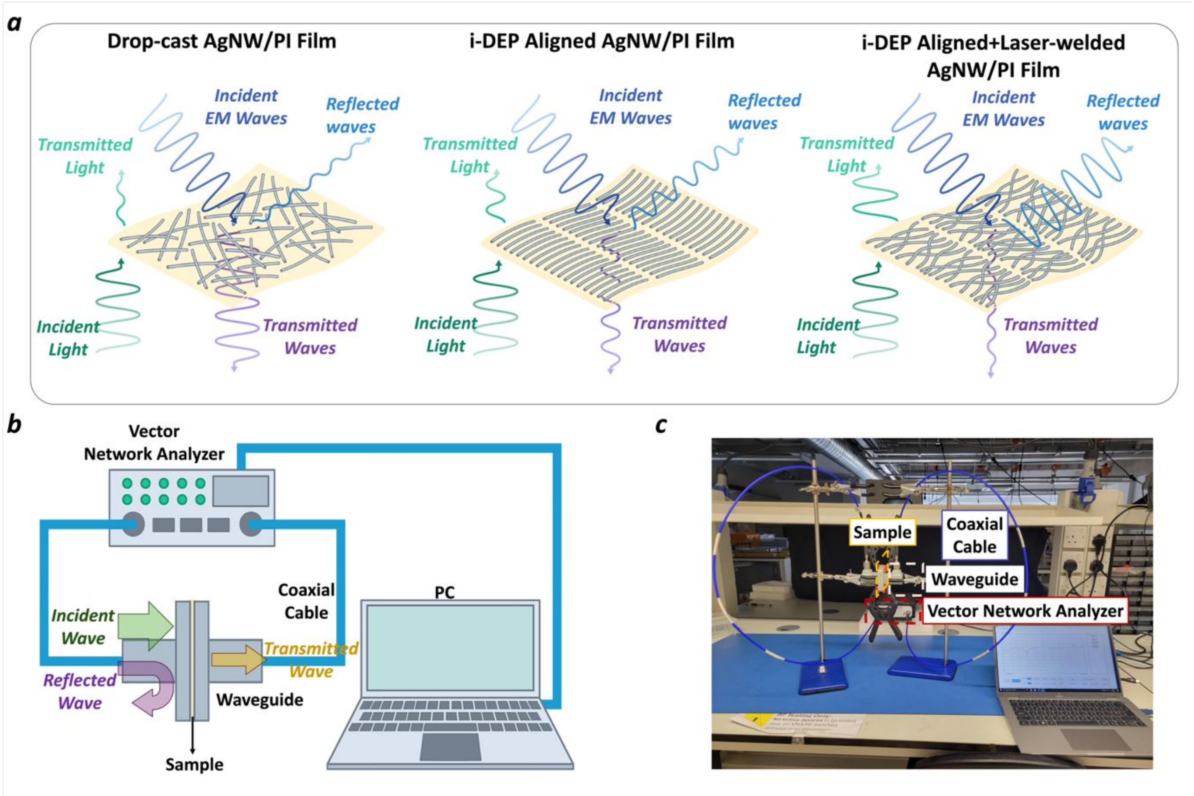

Figure S9. (a) Performance Comparison of EMI shielding materials (shielding effectiveness and optical transmittance) for drop-cast, i-DEP aligned, and i-DEP + laser welded AgNW/PI films. (b-c) Schematic illustration and experimental set-up used for EMI shielding materials characterization.

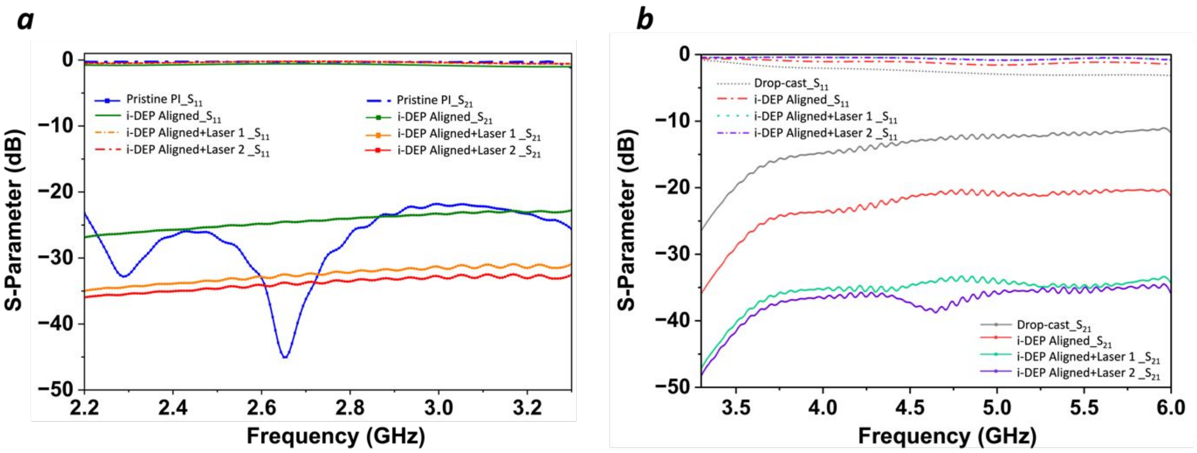

Figure S10. (a-b) Measured S-parameter results of pristine PI, drop-casted AgNW/PI, i-DEP-aligned AgNW/PI, and i-DEP-aligned + laser-welded AgNW/PI films over the 2.2–6 GHz frequency range.

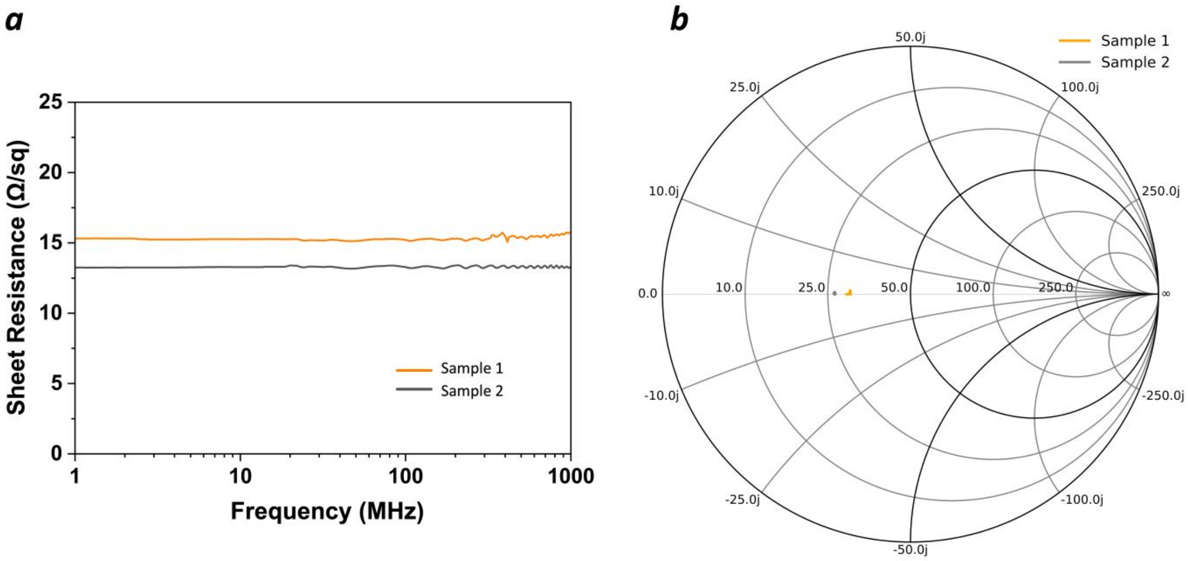

Figure S11. (a) Frequency-dependent sheet resistance of the laser welded i-DEP aligned AgNW/PI film. (b) Smith Chart plots the measured complex impedance of the AgNW/PI films.

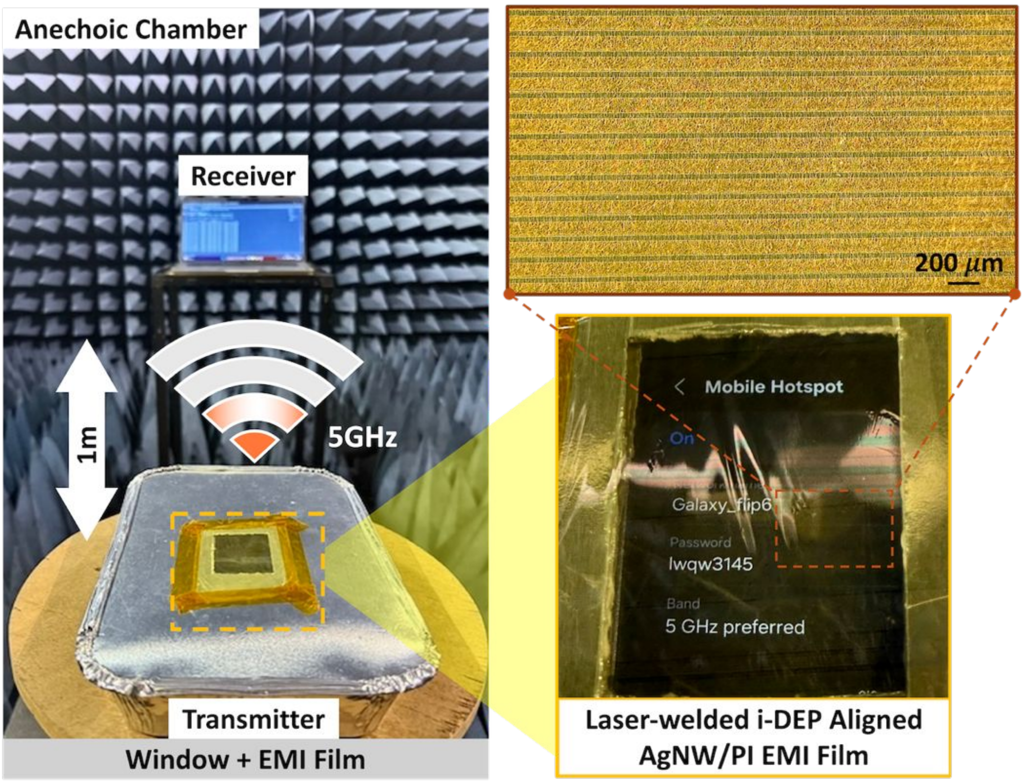

Figure S12. Real-time RSSI measurement setup inside an anechoic chamber, demonstrating a scenario in which an open window is covered with an i-DEP-aligned, laser-welded AgNW/PI film. A smartphone continuously transmits a 5 GHz Wi-Fi signal over a 200-second interval to evaluate signal attenuation. The EMI shielding film remains optically transparent, allowing the smartphone to be visible through it, as shown in the magnified image.

Table SII. Comparison of SE and Optical transparency of State-of-the-Art AgNW-based Films

Material	Transmittance (%)	Shielding Efficiency (dB)	Reference
PES/Silver Nanowires/PET	90	5	[S1]
	85	16	
	81	25	
	76	28	
	70	35	
Wet-sintered AgNW	84.9	20	[S2]
	80.3	30.1	
PAA/AgNW@PI/PAA	82	29	[S3]
	78	33	
	60	38	
	40	47	
PHBV/AgNW	60.5	32.2	[S4]
	74.3	20	
	15	45.9	
	30	41.1	
PVP/AgNW	75	24	[S5]
	80	21	
	85	19	
	92	17.7	
Gelatin/AgNW	72	37.74	[S6]
	75.94	30.07	
	79.7	20.94	
PDMS/AgNW	76.1	25.5	[S7]

	74.9	30.9	
	63.3	35.7	
	53.1	39.1	
Aligned AgNW on PDMS	73.7	32.2	[S8]
Aligned AgNW	72.9	35.2	[S9]
Aligned Ag/Graphene Core-Shell NW	92	36	
Random PEDOT:FeNW Composite	50.84	14.57	[S10]
Aligned PEDOT:FeNW Composite	75.78	19.37	
Network Structure-45 degree aligned PEDOT:FeNW Composite	76.61	33.54	
$Ti_3C_2T_x$ MXene/AgNW (Single Layer)	86.6	23.7	[S11]
$Ti_3C_2T_x$ MXene/AgNW (two-layer)	83	49.2	
$Ti_3C_2T_x$ MXene/AgNW (four-layer)	59	95.6	
MXene / AgNW bilayer	81	24.6	[S12]
(AgNW)@MXene on transparent wood	28.8	44	[S13]
$Ti_3C_2T_x$ MXene / AgNW hybrid conductive network	52.3	32	[S14]
$Ti_3C_2T_x$ MXene / AgNW composite on PU	82.8	27.1	[S15]
**Drop-Cast AgNW**	**5**	**75**	**This Work**
**i-DEP-Aligned AgNW**	**27**	**75**	
**i-DEP-Aligned + Laser Treated AgNW**	**35**	**83.1**	

Supplemental Section Reference:

[S1] Hu, M. Gao, J., Dong, Y., Li, K., Shan, G., Yang, S., and Li, R. K-Y., "Flexible Transparent PES/Silver Nanowires/PET Sandwich-Structured Film for High-Efficiency Electromagnetic Interference Shielding", *Langmuir* 2012, 28 (18), 7101–7106.

[S2] Kim, D. G., Choi, J. H., Choi, D.-K., and Kim, S. W., "Highly Bendable and Durable Transparent Electromagnetic Interference Shielding Film Prepared by Wet Sintering of Silver Nanowires", *ACS Applied Materials & Interfaces*, **2018**, 10 (35), 29730-29740.

[S3] Huang, Z., Xin, Y., Shen, J., Shen, L., Liu, J., Zeng, X., Ling, H., Tu, G., Yang, H., He, D., Hu, B., "Transparent and lightweight electromagnetic shielding film with resistance to harsh conditions through interpenetrating encapsulation of silver nanowires network", *Chemical Engineering Journal*, **2025**, 505, 159382.

[S4] Yang, S., Wang, Y.-Y., Song, Y.-N., Jia, L.-C., Zhong, G.-J., Xu, L., Yan, D.-X., "Ultrathin, flexible and sandwich-structured PHBV/silver nanowire films for high-efficiency electromagnetic interference shielding", *Journal of Materials Chemistry C*, **2021**, 9 (9), 3307-3315.

[S5] Wang, Z., Jiao, B., Qing, Y., Nan, H., Huang, L., Wei, W., Peng, Y., Yuan, F., Dong, H. Hou, X., and Wu, Z., "Flexible and Transparent Ferroferric Oxide-Modified Silver Nanowire Film for Efficient Electromagnetic Interference Shielding", *ACS Applied Materials & Interfaces.*, **2020**, 12 (2), 2826-2834.

[S6] Wang, G., Hao, L., Zhang, X., Tan, S., Zhou, M., Hu, W., and Ji, G., "Flexible and transparent silver nanowires/biopolymer film for high-efficient electromagnetic interference shielding", *Journal of Colloid and Interface Science*, **2022**, 607 (1), 89-99.

[S7] Zhu, M., Yan, X., Li, X., Dai, L., Guo, J., Lei, Y., and Xu, H., "Flexible, Transparent, and Hazy Composite Cellulosic Film with Interconnected Silver Nanowire Networks for EMI Shielding and Joule Heating", *ACS Applied Materials & Interfaces*, **2022**, 14 (40), 45697–45706.

[S8] Feng, Y., Song, J., Han, G., Zhou, B., Liu, C., Shen, C., "Transparent and Stretchable Electromagnetic Interference Shielding Film with Fence-like Aligned Silver Nanowire Conductive Network", *Small Methods*, **2023**, 7 (7), 2201490.

[S9] Zhao, W., Dong, J., Li, Z., Zhou, B., Liu, C., Feng, Y., "Centrifugal Inertia-Induced Directional Alignment of AgNW Network for Preparing Transparent Electromagnetic Interference Shielding Films with Joule Heating Ability", *Advanced Science*, **2024**, 11 (38), 2406758.

[S10] Yang, P.-a., Qu, Z., Ruan, H., Huang, Y., Huang, X., Li, W., Xia, T., Li, R., "Nanostructured Films of Ordered Fe Nanowires for High-Performance Transparent Electromagnetic Interference Shielding", *ACS Applied Nano Materials*, **2023**, 26 (10), 8540-8549.

[S11] Chen, W., Liu, L.-X., Zhang, H.-B., and Yu, Z-Z.,* "Flexible, Transparent, and Conductive Ti3C2Tx MXene–Silver Nanowire Films with Smart Acoustic Sensitivity for

High-Performance Electromagnetic Interference Shielding" ACS Nano, 2020, 14 (12), 16643–16653.

[S12] Jin, M., Chen, W., Liu, L.X., Zhang, H.B., Ye, L., Min, P. and Yu, Z.Z., "Transparent, conductive and flexible MXene grid/silver nanowire hierarchical films for high-performance electromagnetic interference shielding." Journal of Materials Chemistry A, 2022, 10 (27), 14364-14373.

[S13] Cheng, M., Ying, M., Zhao, R., Ji, L., Li, H., Liu, X., Zhang, J., Li, Y., Dong, X. and Zhang, X., "Transparent and flexible electromagnetic interference shielding materials by constructing sandwich AgNW@ MXene/wood composites." ACS nano, 2022, 16 (10), 16996-17007.

[S14] Zhou, B., Su, M., Yang, D., Han, G., Feng, Y., Wang, B., Ma, J., Ma, J., Liu, C. and Shen, C., "Flexible MXene/silver nanowire-based transparent conductive film with electromagnetic interference shielding and electro-photo-thermal performance." ACS applied materials & interfaces, 2020, 12 (36), 40859-40869.

[S15] Wang, Z., Wang, P., Cao, W., Sun, C., Song, Z., Ji, D., Yang, L., Han, J. and Zhu, J., "Robust, transparent, and conductive AgNW/MXene composite polyurethane self-healing film for electromagnetic interference shielding." Journal of Materials Chemistry C, 2022, 10 (45), 17066-17074.
